# Colorism and immigrant earnings in the United States, 2015–2024

**DOI:** 10.3389/fsoc.2024.1494236

**Published:** 2024-11-26

**Authors:** Joni Hersch

**Affiliations:** ^1^Vanderbilt Law School, Vanderbilt University, Nashville, TN, United States; ^2^IZA Institute of Labor Economics, Bonn, Germany

**Keywords:** colorism, race, skin tone discrimination, immigrant, earnings, Current Population Survey, New Immigrant Survey, MENA

## Abstract

Using data from the Current Populations Survey 2015–2024 matched to skin color data in the New Immigrant Survey, this article shows that immigrants from countries with darker skin color face a substantial earnings penalty. The penalty is similar to that found using 2003 data on individual immigrants. Controls for extensive labor market characteristics and race and ethnicity does not eliminate the negative effect of darker skin tone on wages. Color discrimination lawsuits in light of the addition of a Middle Eastern and North African (MENA) reporting category for US government surveys may become more viable.

## 1 Introduction

Using unique data from the 2003 New Immigrant Survey (NIS), I found that new documented immigrants to the US with darker skin color suffered a substantial earnings penalty relative to immigrants who were otherwise comparable on a wide range of individual and labor market characteristics (Hersch, 2008). On average, immigrants with the lightest skin tone earned 17 percent more than otherwise comparable immigrants with the darkest skin tone. After ruling out an array of alternative explanations, discrimination against immigrants with darker skin color appears to be the most likely explanation for the pay penalty.

Although the US is a country founded by and populated by immigrants and their offspring, immigration has always been controversial, and the legal history shows a wide range of laws encouraging or prohibiting immigration from specific countries. Donald Trump made opposition to immigrants—at least those from certain countries—a defining policy of his 2016 presidential campaign and subsequent presidency.^1^

Aside from the political rhetoric, however, whether the penalty to darker skin color has diminished, increased, or remain unchanged over time is an open question. There is abundant data on the labor market outcomes of immigrants, as immigrants are included in and identified in government surveys and in other nationally representative data sets. The main barrier to gaining more recent evidence on the relationship of skin color to immigrants’ economic outcomes is the absence of data on skin color. Since the NIS was fielded in 2003, there has been no new data that reports information on immigrants and skin color.

To overcome this obstacle and provide recent information on the skin color–earnings relationship, I estimate wage equations using the standard methodology widely used in the hedonic wage literature. For information on earnings, individual characteristics, and labor market status, I use data from the Current Population Survey (CPS) for 2015–2024. For information on skin color, I match to individuals in the CPS the average skin color by country of birth and gender calculated from the NIS and reported in Hersch (2008). I include in the wage equations this measure of skin color in addition to extensive individual and work-related characteristics available in the CPS. I note that this approach is feasible only because of the between-country variation in skin color.

Despite the differences in the data sources and time period, I find a pay penalty to immigrants from countries with darker skin tone of a magnitude very close to that in Hersch (2008). The pay penalty is robust to alternative specifications. These findings indicate that overall, time has brought little change in the penalty to darker skin color among immigrants. However, the penalty is more than 20 percent higher during the Trump administration than in the subsequent Biden administration.

## 2 Motivation and empirical specification

### 2.1 Background

It is widely recognized that lighter skin color confers preferential treatment and advantaged outcomes among many dimensions and in almost every culture or region.^2^ There is a large empirical literature documenting advantaged educational, occupational, and earnings outcomes of African Americans with lighter skin tone (e.g., Hughes and Hertel, 1990; Keith and Herring, 1991; Goldsmith et al., 2006, 2007; Hersch, 2006; Monk, 2014; Kreisman and Rangel, 2015). Studies examining other population groups in the US similarly find those with light skin color are advantaged. For example, Mason (2004) and Espino and Franz (2002) document preferential treatment of Hispanics/Latinos in the United States with lighter skin color. Kiang and Takeuchi (2009) find that Filipino Americans residing in San Francisco or Honolulu with darker skin color have lower income.

Studies that examine the relationship between skin color and economic outcomes among immigrants to the US use data from the 2003 New Immigrant Survey (NIS). Hersch (2008, 2011, 2018) documents a substantial pay penalty to immigrants with darker skin color. Frank et al. (2010) shows an earnings penalty to darker skin tone among Latino immigrants. Han (2020) shows that those with darker skin color experienced greater downward occupational mobility upon immigrating to the US and subsequently slower upward mobility. Rosenblum et al. (2016) stratify the NIS sample by region of birth and by self-reported race, finding that the most consequential penalty arises among those darker skinned Latinx immigrants who report their race as White.

### 2.2 Empirical specification

To investigate the relationship between skin color and earnings among immigrants, I estimate a standard log wage equation of the following general form:


(1) lnwage=Xβ+Zδ+γS+ε,

where *wage* is the hourly wage rate and *X* is a vector of individual characteristics that may be related to earnings such as education, age, and time since migration. *Z* is a vector of characteristics related to current employment status such as occupation and union status. These variables are available in the CPS and are defined below. To account for the stratified sampling frame, all estimates are weighted by the earning weight.

The key variable in Equation 1 is skin color *S*, implemented as the average value for the individual’s country and gender. The NIS provides interviewer-assigned measures of skin color for immigrants in the sample. Average skin color varies by race, as expected, but also varies on average by country of origin even among those with the same identified race. To examine the skin color–earnings relationship, I use the methodology common in the hedonic wage literature to estimate wage–job risk tradeoffs. In this literature, the wage equation includes as a regressor the average job risk measure calculated from a source that reports data on job risk (e.g., fatalities, injuries) matched to those with the same characteristics in the data set that reports earnings and other labor market and individual characteristics. Similarly, I assign to individuals in the CPS sample the average skin color calculated from the NIS for the individual’s country of origin and gender. Because skin color is assigned by country, it is not possible to also control for country of origin as would be possible if skin color was individual-specific. I describe the NIS survey and the measures of skin color in the next section.

## 3 Data

### 3.1 Current Population Survey

The data on earnings and individual characteristics is drawn from the CPS for the years 2015 through May 2024.^3^ The CPS is a monthly survey of a representative sample of about 60,000 households. It is the primary government source of data on the unemployment rate, earnings, labor force participation, and other labor market information. Households are surveyed for four consecutive months and then again for four months a year later. In every round, employment status is reported for household members ages 16 and over. Respondents in their fourth and eighth interview are in the “outgoing rotation group” and are asked to report additional information on their employment status including earnings, hours worked, and union status. I restrict the sample to those in the earnings eligible sample. Earnings for those who are self-employed are not available in the monthly CPS (it is available only in the March Annual Social and Economic Supplement). To remain focused on possible employer discrimination and to ensure a large sample size, I use the monthly CPS. Self-employed are not included in the analyses.

### 3.2 Variable definitions

The key outcome variable is the log of the real hourly wage rate. In the CPS, hourly wage is either reported directly for those paid hourly or is calculated as weekly earnings divided by usual hours worked per week.^4^ I standardized the wage rate to January 2024 dollars using the Consumer Price Index for Urban consumers.

The explanatory variables include demographic, employment, and location information. Demographic information includes age, education, race, ethnicity, citizenship status, and years since immigration. Employment information includes occupation, class of worker, union status, and whether paid hourly. Location information includes region of the US and metropolitan location. The specific variables are defined as follows.

Age is reported in years. Education is reported in categories. I assign years of education using the midpoints of the categories. Race and ethnicity are recorded in separate questions. Respondents are asked whether they are of Hispanic, Latino, or Spanish origin (hereafter, Hispanic for brevity). I group race into mutually exclusive categories of single race White, Black, or Asian, and a category for other races, or more than one race reported.^5^ There are few observations in the latter category and few immigrants.

All respondents report their country of birth and their citizenship status. Those who were born in the US, in US outlying areas, or born abroad of US citizen parents are defined as native-born. The remaining respondents are grouped as naturalized citizens or not a citizen. Respondents report the year they immigrated, which is grouped into ranges in the CPS (the ranges are usually two years). I define years since immigration as the difference between the survey year and the top value of the range. This will somewhat underestimate the years since immigration for those who immigrated earlier than the end of the range.

Class of worker is reported as self-employed, private employee, or government employee. Self-employed workers are not considered in the analyses. I create indicator variables for private and government employees. Union is an indicator equal to one for those who are a union member or are covered by a union contract or employee association. I define an indicator for full-time employment for those who usually work full-time (whether or not they did so in the survey week). Occupation is grouped into five categories using the top-level 2010 Census occupation groups (management, professional, and related; service; sales and office; natural resources, construction, and maintenance; production, transportation, and material moving).

To take into account regional and city-size differences in cost of living and population characteristics such as concentration of immigrants, I include indicators for location. Location is identified by indicators for the four broad Census regions (Northeast, Midwest, South, West) and with an indicator for location in a metropolitan area. I also include indicator variables for survey year to account for trends in economic conditions.

The sample is restricted to immigrants who are employed, not self-employed, ages between 18 and 70, with real wages between $4 and $150 per hour in $2024. Appendix A indicates the number of observations eliminated by each restriction. Descriptive statistics overall and by gender for the variables included in the wage equations are reported in Table 1. Column 1 reports statistics for the full sample of immigrants and columns 2 and 3 report these values by gender. The sample size of immigrants is 206,961, with 93,233 women and 113,728 men. For comparison, column 4 reports the corresponding values for native-born US citizens.

**Table 1:** Descriptive statistics for variables in wage regressions: Mean (standard deviation) or percent.

	Immigrant sample			Native-born US sample
	(1)	(2)	(3)	(4)
	All	Women	Men	All
Real hourly wage (2024$)	28.91 (19.40)	26.50 (18.06)	30.76 (20.18)	29.99 (18.35)
Log of real hourly wage (2024$)	3.18 (0.59)	3.10 (0.58)	3.24 (0.59)	3.24 (0.56)
Skin color	4.32 (1.16)	4.13 (1.13)	4.46 (1.16)	–
Exact country match	72.23	71.09	73.11	–
Age	42.77 (12.07)	43.23 (12.08)	42.42 (12.05)	40.83 (13.71)
Female	43.45	100.00	0.00	49.00
Years of education	13.39 (3.90)	13.74 (3.66)	13.13 (4.06)	14.44 (2.34)
Years since immigration	19.08 (12.34)	19.51 (12.30)	18.75 (12.35)	–
Naturalized US citizen	48.09	53.87	43.64	–
Non-citizen	51.91	46.13	56.36	–
Hispanic/Latino (any race)	47.95	43.13	51.65	11.76
Single race White	58.54	54.65	61.53	80.85
Single race Black/African American	11.90	13.43	10.72	13.12
Single race Asian	26.08	28.57	24.16	2.43
Other race or more than one race	3.48	3.35	3.58	3.60
Private employer	91.10	88.38	93.18	83.31
Government employer	8.90	11.62	6.82	16.69
Union or employee association	9.58	10.26	9.07	12.43
Usually full-time	85.64	78.64	91.02	84.11
Paid hourly rate	58.41	61.82	55.80	56.51
Management, professional, and related	34.06	37.31	31.56	42.59
Service	22.31	30.28	16.18	14.89
Sales and office	14.84	19.93	10.94	23.01
Natural resources, construction, and maintenance	13.43	1.98	22.23	7.88
Production, transportation, and material moving	15.36	10.50	19.09	11.64
Metropolitan location	96.14	96.54	95.84	86.23
Northeast	20.92	22.19	19.94	16.95
Midwest	11.91	11.67	12.10	23.67
South	35.14	34.38	35.72	37.75
West	32.03	31.76	32.24	21.63
N	206,961	93,233	113,728	1,218,381

Author’s calculations from the earnings sample of the Current Population Survey 2015–May 2024. Except for skin color, all values are reported in the CPS. Skin color is matched by gender and place of birth from the average skin color for that gender and place of birth calculated using the New Immigrant Survey 2003 and reported in Hersch (2008). The samples are restricted to those ages 18–70, with real hourly wage $4.00–$150.00 (in real January 2024 dollars). All values are weighted by the earnings weight.

As a brief overview of some of the sample characteristics, first compare the overall averages for the immigrant sample reported in column 1 to the corresponding native-born sample in column 4.^6^ Although hourly wages are somewhat higher for the native-born sample, the difference is only about 4 percent and confirms that immigrants’ faster earnings growth leads to rapid convergence (Hersch and Shinall, 2018).

Following the Immigration and Nationality Act of 1965, post-1965 immigrants primarily arrive from Asia and Latin America. This immigration pattern is reflected in the sample characteristics. The Hispanic share of the immigrant sample is 48 percent, in contrast to the 12 percent share in the native-born sample. The share of the immigrant sample reporting their race as Asian is 26 percent, compared to less than 3 percent among the native-born sample. Fifty-nine percent of the full immigrant sample report their race as White. In contrast, 81 percent of the native-born sample report their race as White. A higher share of the native-born sample is employed in management, professional, and related occupations; and in government jobs (which often require US citizenship). Immigrants are more likely to reside in the West and less like to reside in the Midwest relative to the native-born.

Comparing women and men immigrants in columns 2 and 3, the statistics show the usual pattern of lower earnings for women, with women immigrants on average earning about 16 percent less than men. Women are 10 percentage points more likely to be naturalized US citizens. For both men and women, the average duration since immigrating to the US is about 19 years.

### 3.3 New Immigrant Survey skin color measure

The 2003 NIS provides data on a nationally representative sample of immigrants admitted to lawful permanent residence status in 2003 drawn from a sampling frame of US government records (Jasso et al., 2005). Most important for the present purpose is the measure of skin color that was recorded by interviewer observation for 4,652 adult respondents (out of the full sample of 8,573 observations). Skin color is recorded as one of 11 values ranging from zero (albinism) to 10 (darkest possible shade) using a scale designed by Massey and Martin (2003).^7^ Analysis in Hersch (2008) confirms the reliability of the measure. In addition, Hersch (2008) confirms that the skin color scale can be used as a cardinal scale instead of as only an ordinal scale (as might be the case for skin color measures found in surveys with three to five categories such as light, medium, dark; or very light, light, medium, dark, very dark). Because the scale allows a cardinal interpretation, use of the average skin color value is statistically appropriate. Hersch (2008; Table 2) reports average skin color by gender and country or region. For women, average skin color ranges from 1.07 (for countries in the Oceania region) to 7.33 (Nigeria). For men, the range is from 1.96 (Poland) to 8.32 (Nigeria).^8^

**Table 2:** Wage equation estimates for immigrant sample: Dependent variable = ln (real hourly wage).

	(1)	(2)	(3)
Skin color	−0.0336** (0.0011)	−0.0294** (0.0011)	−0.0167** (0.0010)
Age	0.0490** (0.0006)	0.0469** (0.0006)	0.0311** (0.0006)
Age squared/100	−0.0499** (0.0007)	−0.0500** (0.0007)	−0.0318** (0.0006)
Years of education	0.0764** (0.0003)	0.0746** (0.0003)	0.0360** (0.0003)
Female	−0.2096** (0.0024)	−0.2150** (0.0023)	−0.1485** (0.0023)
Years since immigration		0.0028** (0.0001)	0.0018** (0.0001)
Naturalized US citizen		0.0587** (0.0027)	0.0387** (0.0024)
Metropolitan location		0.0934** (0.0050)	0.0809** (0.0045)
Northeast		0.0627** (0.0033)	0.0645** (0.0030)
Midwest		0.0206** (0.0038)	0.0360** (0.0034)
West		0.0894** (0.0028)	0.1013** (0.0025)
Private employer			0.0529** (0.0040)
Union or employee association			0.0878** (0.0038)
Usually full-time			0.1489** (0.0032)
Paid hourly rate			−0.1941** (0.0027)
Management, professional, and related			0.4273** (0.0039)
Service			−0.0937** (0.0031)
Sales and office			0.0447** (0.0037)
Natural resources, construction, and maintenance			0.0967** (0.0037)
Constant	1.2300** (0.0147)	1.1147** (0.0154)	1.6867** (0.0153)
Adjusted R^2^	0.32	0.33	0.48
N	206,961	206,961	206,961

Author’s calculations from the earnings sample of the Current Population Survey 2015–May 2024 matched to skin color measures calculated from the New Immigrant Survey 2003. The samples are restricted to those ages 18–70, with real hourly wage $4.00–$150.00 (in real January 2024 dollars). Indicators for survey year are included in all regressions but these coefficients are not reported. Robust standard errors are reported in parentheses. All values are weighted by the earnings weight. ** *p* < 0.01.

Country of birth is recorded from US government records for all sample members in the NIS, with 22 countries separately identified in the public use data and the remaining countries grouped into broad regions such as Middle East and North Africa. I assign skin color to individuals in the CPS data by matching to the average skin color corresponding to the individual’s country of birth and gender reported in Hersch (2008; Table 2).^9^ Seventy percent of the NIS sample are from the 22 separately identified countries. As indicated in Table 1, 72 percent of the CPS sample are from these same countries. I assign the average for the corresponding region to observations in which the skin color measure is based on the regions. I report earnings equations both including and excluding observations in which the country is not specifically identified in the average skin color measures.

There are two methodological features worth noting. First, because skin color is matched at the country level, it is not possible to also control for country of birth in the regressions. Second, this approach would not be feasible to study, for example, the role of skin color for Black Americans in the US because the average would be the same for all Black Americans.^10^

It is worth pausing here to consider the sources and consequences of possible measurement error in the skin color measure. There are three sources of measurement error: interviewer ratings, assignment of the gender and country average to individuals, and assignment of regional averages when the skin color measure for an individual country is not available. But it is important to keep in mind that as long as any measurement error is random, the estimated effect of skin color on earnings would be biased toward zero. In that case, the true effect of skin color on earnings would be underestimated.

By considering each of the three possible sources of measurement error, it is evident that any measurement error is random. First, although skin color is assigned by interviewer observation in the NIS, systemic measurement error appears to be unlikely (Hersch, 2008), indicating that the average skin color measure is an unbiased estimate of skin color by gender and country. Second, because the average skin color measure is an unbiased estimate of skin color, assigning this average measure to individuals does not introduce bias. The third possible source of measurement error arises from assigning regional averages for observations in which the country is not identified in the NIS. There is likely to be greater random measurement error in average skin color associated with these observations. In this case, we would expect the downward bias associated with random measurement error to be exacerbated.

It is also worth recognizing that in addition to any attenuation bias associated with random measurement error, because skin color for each individual is assigned as the country and gender average, and there are only 22 identified countries, there is less variation in the skin color measure assigned to individuals than there would be if the measure of skin color was individual-specific. The more-limited variation automatically leads to larger standard errors and a lower probability of finding statistically significant effects than if there was greater variation.

Given these known data limitations, we can summarize how to interpret the estimates. Finding nonsignificant effects of skin color does not mean that there is not a relationship. But finding significant effects is strong evidence in support of a skin color effect, and one that is likely a lower bound on the magnitude.

## 4 Results

The wage equation estimation proceeds in stages, starting with a baseline specification that includes only pre-market characteristics and consecutively including additional variables that may be in part related to any possible skin tone discrimination. To the extent that skin color is correlated with the variables in the wage equation, the magnitude of the coefficient on skin color will be reduced. And to the extent that these characteristics are themselves associated with discrimination, the coefficient on skin color will not capture the full effect of any discrimination and will instead be an underestimate of the magnitude.

In the following regressions, the dependent variable is the log of real hourly wage. The specifications reported in Table 2 pool men and women and include an indicator variable for gender. Estimates reported in Tables 3 and 4 examine possible gender differences in the relation between skin color and wages as well as alternative specifications and sample periods.

**Table 3:** Wage equation estimates by gender and including race and ethnicity: Dependent variable = ln (real hourly wage).

	(1)	(2)	(3)
	Women	Men	Including race and ethnicity
Skin color	−0.0201** (0.0016)	−0.0357** (0.0015)	−0.0057** (0.0015)
Age	0.0392** (0.0009)	0.0528** (0.0008)	0.0469** (0.0006)
Age squared/100	−0.0431** (0.0010)	−0.0550** (0.0010)	−0.0507** (0.0007)
Years of education	0.0797** (0.0005)	0.0711** (0.0004)	0.0658** (0.0004)
Years since immigration	0.0037** (0.0002)	0.0019** (0.0002)	0.0039** (0.0001)
Naturalized US citizen	0.0660** (0.0038)	0.0529** (0.0037)	0.0398** (0.0027)
Metropolitan location	0.0883** (0.0074)	0.0972** (0.0066)	0.0796** (0.0049)
Northeast	0.0791** (0.0048)	0.0501** (0.0047)	0.0450** (0.0033)
Midwest	0.0408** (0.0057)	0.0062 (0.0051)	−0.0036 (0.0038)
West	0.1102** (0.0042)	0.0749** (0.0038)	0.0673** (0.0028)
Female			−0.2111** (0.0024)
Hispanic/Latino (any race)			−0.1467** (0.0037)
Single race Black/African American			−0.1293** (0.0060)
Single race Asian			0.0490** (0.0039)
Other race or more than one race			−0.0203** (0.0066)
Constant	0.9616** (0.0226)	1.0557** (0.0208)	1.2315** (0.0160)
Adjusted R^2^	0.32	0.32	0.34
N	93,233	113,728	206,961

Author’s calculations from the earnings sample of the Current Population Survey 2015–May 2024 matched to skin color measures calculated from the New Immigrant Survey 2003. The samples are restricted to those ages 18–70, with real hourly wage $4.00–$150.00 (in real January 2024 dollars). Indicators for survey year are included in all regressions but these coefficients are not reported. Robust standard errors are reported in parentheses. All values are weighted by the earnings weight. ** *p* < 0.01.

**Table 4:** Alternative samples and specifications: Dependent variable = ln (real hourly wage).

Row number	Sample or specification	Skin color coefficient (standard error)	*N*
1	Baseline Table 2, column 2	−0.0294 (0.0011)	206,961
2	Exact country match	−0.2577 (0.0015)	147,976
3	Clustered and robust standard errors	−0.0294 (0.0121)	206,961
4	Survey years 2015–2019 (Pre-COVID)	−0.0316 (0.0014)	119,692
5	Survey years 2020 and 2021 (COVID)	−0.0281 (0.0025)	39,031
6	Survey years 2022–May 2024 (Post-COVID)	−0.0260 (0.0022)	48,238
7	Less than 10 years since immigration	−0.0289 (0.0020)	52,044
8	10–20 years since immigration	−0.0288 (0.0019)	67,569
9	More than 20 years since immigration	−0.0303 (0.0018)	87,348
10	Trump administration 2017–2020	−0.0321 (0.0017)	90,324
11	Biden administration 2021–2024	−0.0259 (0.0019)	68,243

Author’s calculations from the earnings sample of the Current Population Survey 2015–May 2024 matched to skin color measures calculated from the New Immigrant Survey 2003. The samples are restricted to those ages 18–70, with real hourly wage $4.00–$150.00 (in real January 2024 dollars). All equations include age and its square, education, gender, years since immigration, citizenship status, metropolitan location, region, and survey year. Robust standard errors are reported in parentheses. All values are weighted by the earnings weight. All coefficients are statistically significant with *p* < 0.01.

In Table 2, column 1 controls only for skin color, age and its square, years of education, an indicator variable for gender, and indicator variables for survey year. This is a standard human capital specification of the wage equation and allows comparison to estimates in the literature. Column 2 adds variables relevant to immigrant status, specifically years since immigration, an indicator variable for whether the individual is a naturalized US citizen (versus non-citizen), and location characteristics (metropolitan location and region of country). It is possible that these characteristics are influenced to some extent by skin tone discrimination. For instance, whether an individual enters the US with an employment visa may be related to skin color if there is bias originating from sponsoring employers. And the time it takes to receive legal entry status or to become a naturalized citizen may be related to the skin color associated with the originating country. In addition, location may be related to skin color because immigrants may select their location upon entering the US based on various factors including labor market conditions that may be more or less favorable to immigrants of different skin color.

Column 3 includes additional variables associated with current employment. Specifically, this equation adds indicator variables for private employer (versus government), union status, full-time employment, whether paid hourly, and broad occupational category (with production and transportation the reference category). Characteristics that are associated with current employment may themselves be influenced by discrimination. If so, the coefficient on skin color will be smaller in column 3, but it does not necessarily imply an absence of color discrimination but may instead indicate that any colorism is manifested through sorting in the labor market.

Before discussing the skin color estimates, it is useful to consider the other variables in the wage equations. The regressions reported in Table 2 show the conventional relationships between age, education, and gender. Wages rise with age at a decreasing rate, wages are higher for those with more education, and women earn substantially less than men. The estimates reported in columns 1 and 2 are very similar and show that wages peak when individuals are around 47 years of age, each additional year of education is associated with about 7.5 percent higher wages, and women have earnings lower than men by about 21 log points. The results in column 2 show that each additional year since immigration is associated with 0.3 percent higher wages, and wages are about 6 percent higher for naturalized US citizens relative to noncitizens. Wages vary by location and are higher in metropolitan areas and lower in the south than in the other three regions, a pattern consistently seen in earnings regressions.

Inclusion of current employment characteristics in column 3 reduces the magnitudes of the coefficients on age, education, and gender, meaning that current employment characteristics are correlated with these characteristics. For instance, part of any wage advantage to more education will be captured by employment in a higher-paying occupation, and management, professional, and related occupations show a high premium of 43 log points relative to those in production and transportation occupations. Similarly, a comparison of columns 2 and 3 shows that inclusion of current employment characteristics reduces the magnitudes of some of the coefficients, but the patterns and statistical significance are unchanged. The current employment variables included only in the column 3 estimates show the typical pattern and magnitude, with wages higher for those with union status, full-time employment, and paid a salary rather than an hourly rate.

Most important is the relation between skin color and wage. Column 1 shows a statistically significant penalty to darker skin color. The magnitude in column 1 shows that an additional unit of skin color darkness on the 11-point scale lowers wages by 3.4 percent. This magnitude is very similar to the coefficient on skin color of 3.1 percent reported in Hersch (2008; Table 3, column 1) that is calculated using individual-level data from the NIS. The estimates in column 2 show a slightly smaller but similar skin color penalty of 3 percent. These estimates indicate that a one-standard deviation increase in a country’s average skin color scale lowers wages on average by 3.4 to 3.9 percent.

Inclusion of current employment characteristics in column 3 cuts the magnitude of the skin color penalty in half, but the coefficient remains statistically significant and substantial at 1.7 percent. The reduction in the magnitude means that part of the skin color penalty arises from a correlation of skin color with current employment characteristics. Because current employment characteristics may be subject to skin color discrimination, it is notable that darker skin color continues to show a negative effect on wage.

Tables 3 and 4 report estimates from additional specifications and samples. All of these estimates are based on the Table 2, column 2 specification as a middle-ground between the most restricted estimates that control only for age, education, and gender, and the most expansive set of estimates that include current employment characteristics. Specifically, these equations control for age and its square, education, gender, years since immigration, citizenship status, metropolitan location, region, and survey year.

To consider whether there are gender differences in the skin color penalty, Table 3, columns 1 and 2 report the estimates stratified by gender. There is reason to examine whether the penalty to darker skin tone differs by gender. There is substantial evidence suggesting that a darker skin tone is more disadvantageous for women than for men (Hunter, 1998, 2005). By contrast, Hersch (2008) found no gender difference in the relation of skin color to wages among immigrants. The estimates in Table 3 show a lower penalty for women than for men, with an additional unit of skin color darkness on the 11-point scale lowering wages by 2.0 percent for women and 3.6 percent for men. The larger penalty for men is puzzling. Perhaps recent societal trends have eroded the disadvantages faced by women with darker skin color relative to men, but further investigation is warranted.

Table 3, column 3 presents estimates controlling for ethnicity and race in addition to skin color. Because of the high correlation between skin color and race, a key concern in any analysis of whether skin color influences earnings is whether we are actually estimating only a race effect instead of a combination effect of race and color, or an effect of color only. Earnings disparities on the basis of ethnicity and race are widely established. In the labor market as a whole, relative to comparable White workers, estimates typically show lower earnings for Black and Hispanic workers and similar or higher earnings for Asian workers.

The estimates in Table 3 show the typical earnings pattern with respect to ethnicity and race observed in the US labor market as a whole. Relative to comparable immigrant White workers, Black and Hispanic workers have lower hourly wages by 13 to 15 log points, and Asian workers have wages higher by about 5 log points. Most notable is that even controlling for ethnicity and race, the estimates continue to show a negative and statistically significant penalty to darker skin color. The magnitude of the coefficient is smaller but shows that an extra unit on the 11-point skin color scale lowers wages by 0.6 percent on average.

These estimates document that there is an additional penalty for immigrants from countries with darker skin color beyond any penalties associated with ethnicity or race. This finding is consistent with that found in Hersch (2008) using individual data from the NIS, which shows that the penalty to darker skin color persists even with controls for ethnicity, race, and country of birth.

## 5 Alternative specifications

Estimates from alternative specifications and samples are summarized in Table 4. All estimates reported in this table are based on the specification reported in Table 2, column 2. Row 1 repeats the coefficient on skin color reported in Table 2 for reference. Row 2 restricts the sample to those in which the specific country is identified and excludes those observations in which skin color is assigned by the region. Because all individuals within a country of origin and gender group are assigned the same skin color value, row 3 reports standard errors clustered by country of origin and gender to account for the correlation of the error term for observations within the same country and gender.

To examine any possible effects related to the COVID pandemic, rows 4–6 divide the sample into three periods. Employment status, remote work, and survey responses rates were affected by the pandemic. The before-COVID period includes the years 2015–2019. I define the COVID period as the years 2020 and 2021. Although the federal public health emergency did not officially end until May 2023, I consider the period beginning in 2022 through the end of the analysis sample in May 2024 as the post-COVID period.

To investigate whether time in the US since migration diminishes any skin color effect, I report in rows 7–9 estimates dividing the sample into three periods: those that migrated within 10 years, those that migrated between 10 and 20 years ago, and those who migrated more than 20 years ago. This extends the analyses in Hersch (2011, 2018) which examine whether more time in the US reduced the skin color disparity. By construction of the NIS survey, all immigrants in the survey achieved legal status in 2003. This means that the time in the US before achieving legal status was usually fairly limited for most of the sample members. The CPS data allows examining more variation in time in the US.

Rows 10 and 11 provide estimates divided into the years 2017–2020 corresponding to the Trump administration and the years beginning in 2021 corresponding to the Biden administration.

In Table 4, only the coefficient on skin color is reported for each alternative sample or specification. Notably, the coefficient on skin color remains negative and statistically significant in each specification, and most of the coefficients are similar across all specifications. The magnitude is somewhat smaller in the sample in which skin color is assigned with an exact country match instead of either an exact match or a regional match reported in row 2. As typically found when clustering, the standard error in the row 3 estimate is much larger than in estimates without clustering, but the coefficient remains statistically significant (*p*-value = 0.016). Division into COVID periods had little effect on the magnitude.

Even though longer residence in the US could be expected to mitigate any skin tone effect, the results show that on average, the penalty to darker skin color did not decline with longer duration in the US. This is consistent with the results from both the NIS reinterview survey conducted four years after the initial survey (Hersch, 2018) and from the sample of immigrant spouses who may have had longer legal status in the US than the sampled respondent to the NIS (Hersch, 2011).

Also of interest is the comparison of the coefficients in rows 10 and 11, which are based on dividing the years into the Trump administration and the Biden administration. The coefficient on skin color is −0.0321 during the Trump years and is −0.0259 during the Biden years. The coefficients are significantly different at the 1 percent level and show a penalty that is 24 percent higher during the Trump years than the Biden years.

## 6 Discussion

It is widely recognized that across most cultures, regions, and over time, lighter skin tone confers social and economic advantages. This paper estimates wage equations using data from the CPS for the years 2015 through 2024 matched to measures of skin color calculated from the NIS. The wage regressions show that on average, immigrants to the US from countries with darker skin color continue to suffer a substantial pay penalty, with a magnitude similar to that found in studies based on the 2003 and 2007 NIS. A one-standard deviation increase in a country’s average skin color darkness lowers wages on average by 3.4 to 3.9 percent. Inclusion of characteristics associated with current employment that may themselves be subject to skin color discrimination reduces the wage penalty, but the penalty remains statistically significant and indicates that wages are lower by 1.94 percent with a one-standard deviation increase in the skin color scale. The penalty to darker skin color is not merely picking up any penalties associated with race or ethnicity, as the penalty to darker skin color remains statistically significant with inclusion of race and ethnicity indicators in the regressions.

Although longer time in the US might mitigate any penalty to darker skin color, for example, through assimilation and mobility in the US labor market and by improvement in English language proficiency, the penalty is not mitigated by duration of residence in the US. However, political winds seem to have an impact on the penalty to darker skin color, as the penalty was 24 percent larger during the Trump presidential administration than in the Biden administration.

One possible explanation for lower wages for those from countries with darker average skin color could be that darker skin tone is associated with undocumented status. Even if wrong, inferences about possible undocumented status could lead to worse job offers and lower pay. But it is unlikely that inferences about documented status is the full explanation. An experimental study shows that even if an immigrant is known to be undocumented, White observers have a more favorable view of those who are depicted to have lighter skin and stereotypically Eurocentric features (Ostfeld, 2017).

Evidence of differential economic outcomes on the basis of skin tone potentially has bearing on employment discrimination lawsuits. Title VII of the Civil Rights Act of 1964 prohibits employment discrimination on the basis of race, color, religion, sex, and national origin. Although there is a substantial body of research documenting discrimination on the basis of skin tone, relatively few legal claims allege color discrimination alone but instead cite more than one basis such as color and race, or color and national origin (Hersch, 2012). Furthermore, courts often dismiss the color claim, subsuming the color claim into a race or national origin claim given the facts of the case.^11^ Although Title VII does not prohibit claims of discrimination between parties of the same identifiable race, there are few claims of this type compared to the number of cases involving parties of different races, and courts are typically skeptical of intra-racial claims.

But legal charges of color discrimination are likely to become more frequent in the future. Demographic trends indicate greater skin tone diversity among the US population as the immigrant share of the population and the share of children born to parents of different races or ethnicities increases. Color discrimination charges have substantially increased over time in number and as a share of discrimination charges filed with the Equal Employment Opportunity Commission. In FY 1997, there were 762 charges of color discrimination, representing 0.9 percent of all claims. In FY 2023, there were 5,819 claims, representing 7.2 percent of all claims. For comparison, the share of claims of national origin discrimination was 8.3 percent in FY 1997 and 8.6 percent in FY 2023.^12^

How race and ethnicity is categorized by the government may have bearing on the viability of color discrimination claims. The US Office of Management and Budget (OMB) sets the standards for racial and ethnicity classifications used in government statistical surveys. In the 1997 OMB standard which is currently used, race and Hispanic ethnicity are treated as separate concepts and asked in two questions, with the race question allowing for the first time reporting of more than one race. In the new 2024 OMB standard, race and ethnicity is asked in a single question and a new, separate category for Middle Eastern and North African (typically referred to as “MENA”) has been added to the five racial categories in the 1997 standard. Research from the Bureau of Labor Statistics shows that those who will now indicate MENA would likely have indicated White previously (Loewenstein et al., 2024). While it has been difficult to bring discrimination claims against individuals of the same identified race, under the new classifications, claims of color discrimination among those in the MENA category may be more viable.

## Data Availability

Data necessary to replicate the results of this article are publicly available from IPUMS. See Flood et al. (2023).

## Footnotes

^1^In January 2018, then-president Donald Trump questioned why the US would accept more immigrants from Haiti and “shithole countries” in Africa rather than places like Norway. See, for example, Julie Hirschfeld Davis, Sheryl Gay Stolberg, and Thomas Kaplan, “Trump alarms lawmakers with disparaging words for Haiti and Africa,” *New York Times*, January 11, 2018, https://www.nytimes.com/2018/01/11/us/politics/trump-shithole-countries.html.

^2^Seminal work in this area includes Russell et al. (1992), Hall (1995), Hunter (2005), and Jablonski (2006). Dixon and Telles (2017) provide an excellent survey of the literature related to colorism.

^3^I use CPS data available from IPUMS (Flood et al., 2023). IPUMS CPS harmonizes microdata from the monthly CPS over time, and some variables are recoded from the original CPS.

^4^For those observations who are not paid an hourly rate and who report that their usual hours vary, I calculate their hourly wage based on the hours they reported working in the previous week. For the few respondents who did not report hours for either question, I use information on whether they usually work a full-time or part-time schedule. The modal value of hours worked is 40 for full-time workers and is 20 for part-time workers, so I calculate hourly wage for those respondents not reporting specific hours by dividing by the modal value for their work hours status.

^5^The other single race categories are American Indian/Aleut/Eskimo, Asian or Pacific Islander, Hawaiian/Pacific Islander only, and other single race (not specified). There are too few immigrants in these groups to analyze separately, so I group these observations together into the ‘other’ category.

^6^Because of the large number of observations in all samples, even small differences are usually statistically significant.

^7^The Massey and Martin skin color scale shows a series of hands increasing in darkness numbered from one to 10, but the value zero was used by some interviewers to indicate the lightest possible color (or albinism), making the scale an 11-point scale.

^8^Using the sample of US native-born spouses of the immigrants in the NIS sample, Hersch (2011) calculates an average skin color value of 2.71.

^9^As noted earlier, this approach of matching an average characteristic calculated in one data set to individual observations in a second data set that includes labor market information is standard in the hedonic wage literature.

^10^Stratification by region of birth or race as in Rosenblum et al. (2016) is also not desirable in this analysis because there is little variation in average skin color on each of these dimensions. In addition, there are two conceptual arguments against stratifying by region or race in an analysis of skin color and earnings. First, stratification implies that the labor market is comprised of segmented jobs and that workers of different national origins or races are not competing in the same labor market. For example, stratification by race implies that there are “Black jobs,” “White jobs,” and so forth. Second, among the dominant theories explaining disadvantage to those with darker skin tone is the concept of a skin color hierarchy rather than one based on racial categories (e.g., Bonilla-Silva and Dietrich, 2009).

^11^See Banks (2000), Jones (2000), and Nance (2005) for a description and analysis of the legal environment of color discrimination claims under Title VII.

^12^EEOC Table E1a. Charge receipts by basis or statutes (all statutes) FY 1997–FY 2023. Available at https://www.eeoc.gov/data/enforcement-and-litigation-statistics-0.

## Appendix

**Table A1:** Construction of samples used in wage regressions: Monthly CPS 2015–2024.

	Net number affected	Number remaining
Initial earnings eligible sample		1,550,241
Not an immigrant	1,324,405	225,836
Under age 18	856	224,980
Over age 70	3,683	221,297
Missing hourly wage	12,118	209,179
Real hourly wage < $4.00 (2024$)	1,037	208,142
Real hourly wage > $150 (2024$)	600	207,542
Skin color measure not available	581	206,961
Skin color measure not exact country match	58,985	147,976

Source: earnings sample of the monthly Current Population Survey 2015–May 2024. See Flood et al. (2023).
